# Supplementary material for: Identification of Freezing-Responsive microRNAs and Their Targets in Chinese Jujube by Small RNA and Degradome Sequencing
Source: Int J Mol Sci. 2026 Sep 4;27(17):7912. doi: 10.3390/ijms27177912 (PMC13566890; doi:10.3390/ijms27177912)
Supplement: Supplementary file 1 [file ijms-27-07912-s001.zip › Supplementary Material S2/novel_miR_134.pdf]

Provisional ID	:	novel_miR_134
Score total	:	0.5
Score for star read(s)	:	-1.3
Score for read counts	:	0
Score for mfe	:	0.2
Score for randfold	:	1.6
Score for cons. seed	:	
Total read count	:	47
Mature read count	:	47
Loop read count	:	0
Star read count	:	0

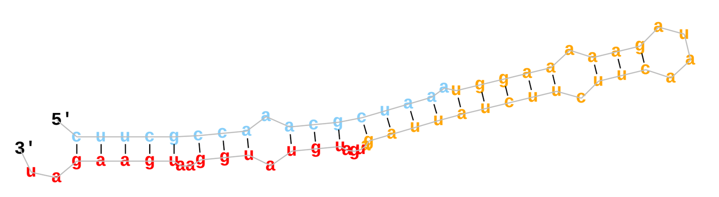

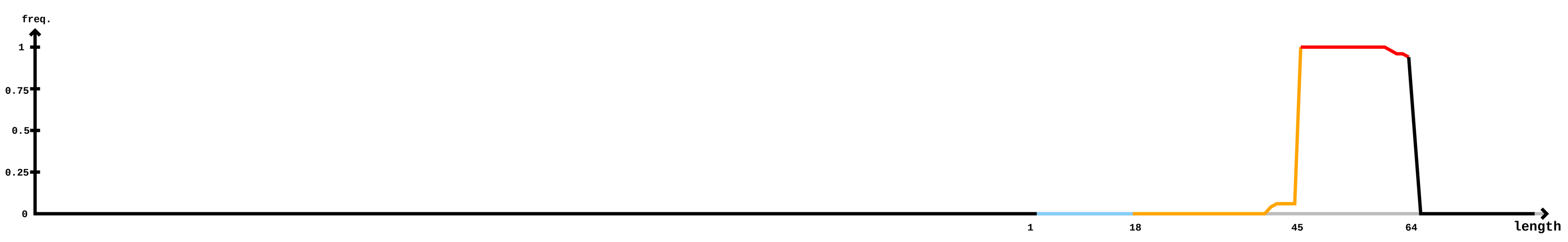

Star Mature

```
5'- aaaauaauucuugagaaaaugauuaaaucgauaaagaaaagagcuucuguagaacaaggccuugaccuggaucaguugcaugguucuguagaacaaggcccugacguagaucagcugcaggguucuguaagacaaggccuuaaccuagaucaguugcaaaacucucuucgccaaacgcuaaauggaaaaagauaacuucuucuauuagaugauguauggaaugaagauagagaaaaauggccuaaauuaa -3'   exp
    .......(((((.....................))))).((((((.((((((.....(((((((..((((((((......))))))...))..)))))))((((......))))))))))))))))........(((((.((((........)))).....((((((((((((.(((((((.(((((.(((....))).)))))))))....))).)))..))))))..))).....))))).......   reads   mm   sample
    ......................................................................................................................................................................................Cuagaugauguauggaauga.........................   1   1   LT-2
    ......................................................................................................................................................................................uuUgaugauguauggaaugaa........................   1   1   LT-1
    .......................................................................................................................................................................................uagaugauguauggaaugaaga......................   1   0   LT-1
    ...........................................................................................................................................................................................ugauguauggaaugaagau......................   20   0   LCK-1
    ...........................................................................................................................................................................................ugauguCuggaaugaagau......................   1   1   LCK-2
    ...........................................................................................................................................................................................ugauguauggaaugaagau......................   26   0   LCK-2
```
